# Falls and mobility in Parkinson's disease: protocol for a randomised controlled clinical trial

**DOI:** 10.1186/1471-2377-11-93

**Published:** 2011-07-31

**Authors:** Meg E Morris, Hylton B Menz, Jennifer L McGinley, Frances E Huxham, Anna T Murphy, Robert Iansek, Mary Danoudis, Sze-Ee Soh, David Kelly, Jennifer J Watts

**Affiliations:** 1 Melbourne School of Health Sciences, The University of Melbourne, 3010, Melbourne, Australia; 2 Musculoskeletal Research Centre, Faculty of Health Sciences, La Trobe University, 3086, Australia; 3 National Parkinson Foundation Center for Excellence, Clinical Research Centre for Movement Disorders and Gait and Victorian Comprehensive Parkinson's Program, Kingston Centre, Warrigal Rd, Cheltenham, 3092, Australia; 4 Centre for Health Economics, Monash University, Building 75, Clayton, 3168, Melbourne, Australia

## Abstract

**Background:**

Although physical therapy and falls prevention education are argued to reduce falls and disability in people with idiopathic Parkinson's disease, this has not yet been confirmed with a large scale randomised controlled clinical trial. The study will investigate the effects on falls, mobility and quality of life of (i) movement strategy training combined with falls prevention education, (ii) progressive resistance strength training combined with falls prevention education, (iii) a generic life-skills social program (control group).

**Methods/Design:**

People with idiopathic Parkinson's disease who live at home will be recruited and randomly allocated to one of three groups. Each person shall receive therapy in an out-patient setting in groups of 3-4. Each group shall be scheduled to meet once per week for 2 hours for 8 consecutive weeks. All participants will also have a structured 2 hour home practice program for each week during the 8 week intervention phase. Assessments will occur before therapy, after the 8 week therapy program, and at 3 and 12 months after the intervention. A falls calendar will be kept by each participant for 12 months after outpatient therapy.

Consistent with the recommendations of the Prevention of Falls Network Europe group, three falls variables will be used as the primary outcome measures: the number of fallers, the number of multiple fallers and the falls rate. In addition to quantifying falls, we shall measure mobility, activity limitations and quality of life as secondary outcomes.

**Discussion:**

This study has the potential to determine whether outpatient movement strategy training combined with falls prevention education or progressive resistance strength training combined with falls prevention education are effective for reducing falls and improving mobility and life quality in people with Parkinson's disease who live at home.

**Trial registration:**

Australia and New Zealand Clinical Trials Register (ANZCTR): ACTRN12606000344594

## Background

Falls are common in people with idiopathic Parkinson's disease (PD) and fall related injuries can be associated with immobility and reduced quality of life. Many people with PD experience difficulties walking and balancing as the disease progresses and this can compromise their ability to participate in work, family, community and social activities [1,2]. Previous research suggests that more than half of people who are diagnosed with PD experience one or more falls in a given 12 month period, compared to 30% of older adults who live in the community [3-8]. The extent to which falls in people with PD are related to hypokinesia, dyskinesa, postural instability, rigidity, weakness, cognitive impairment or medication remains unclear. The associated burden of disease arising from falls and immobility can impact adversely on individuals, their families, the healthcare system and society.

At the present time there is no known cure for idiopathic PD. Pharmacological therapy currently provides the most effective symptomatic treatment for many movement disorders [9]. Nevertheless freezing of gait, postural instability and hypokinesia have only a limited or short-lived response to PD medication in many individuals [10]. As the disease progresses, PD medications are adjusted in response to changes in symptoms [11]. Despite the best medical management, motor fluctuations and movement disorders can recur due to progressive cell loss in the substantia nigra pars compacta in the brainstem and disruption to neural connections to the frontal lobes, cerebellum and other regions of the brain [12]. For these reasons, pharmacological management is often augmented by physical therapy and falls education [13].

There are two main approaches to physical rehabilitation for people with PD. Movement strategy training (MST) teaches individuals how to cope with their movement disorders by using attention, cues, environmental adaptation, part practice and mental rehearsal [14]. Rather than regulating well learned movements automatically through the defective basal ganglia circuitry, MST aims to train people to use the frontal cortex in the initiation and execution of movements and functional activities. Motor performance is optimised by breaking down complex movement sequences into small parts and by teaching people to focus their attention on each part prior to "whole" task practice [13]. Mental rehearsal of forthcoming movements, focussing on the movement while it is occurring and the use of visual or auditory cues to guide motor performance are additional strategies [13,15]. Other "tricks" include performing the movement in a different way (e.g., running or dance steps instead of walking) and using different intent (e.g., thinking about getting to the fridge to get the milk rather than thinking of trying to walk).

The other key approach to improving movement in people with PD is progressive resistance strength training (PST) [16-20]. People with PD can become weak and de-conditioned due to inactivity, disuse and reduced physical activity associated with hypokinesia and ageing [17,18]. There is preliminary evidence that PST might improve muscle strength [18,21,22] with associated improvements in balance [21] and walking [18,19] in some people. The extent to which PST prevents falls in people with PD remains unclear.

Education packages that provide advice about the predictors of falls and how to prevent them are also thought to be effective in reducing falls, either as a single intervention or as part of a multifaceted treatment package [23-26]. Although a range of approaches and methods of delivery have been used, these packages generally aim to increase awareness of a person's risk of, and risk factors for, falling, as well as to help them identify and access resources to undertake appropriate interventions.

The primary aim of the current study is to conduct the first large scale community based trial to investigate if outpatient physical therapy programs comprising either (i) MST combined with falls education or (ii) PST combined with falls education are more effective than a control group that receives a generic "life-skills" program that does not contain any information or advice about gait, balance, falls, exercise or mobility. We hypothesise that participation in physical therapy directed to either MST or PST combined with falls education will reduce falls relative to participation in the control group. Participation in an active therapy group is also predicted to provide significant improvements in mobility and quality of life, which are not expected to occur for the control group. The anticipated reduction in falls rate in the therapy groups is likely to be accompanied by cost benefits, as we have described in an earlier paper [27].

## Methods

### Design

A single blind, parallel-group randomised controlled clinical trial (RCT) design will be used with two intervention groups and one control group. Because the literature at the time of study design did not provide evidence on whether movement strategies were superior to strengthening, we included one group receiving MST and another receiving PST. To reflect clinical practice, each of these groups will also receive a falls prevention education program [28]. A control group that does not include physical therapy or falls education will be used to determine the relative effectiveness of each therapy approach. Ethical approval to conduct the study has been gained from the University of Melbourne Health Sciences Human Ethics Sub-Committee (0828579). Written consent for publication will be obtained from the participant or their relative.

### Participants

Community dwelling people with PD will be recruited for the trial from outpatient movement disorder clinics, community-based rehabilitation programs, PD support groups and private neurologists in the Melbourne metropolitan area. Participants will also be recruited from the local community through advertisements in local and metropolitan newspapers and newsletters distributed by Parkinson's Victoria, Australia. Potential participants will be provided with an information pack describing the study, and invited to return an 'expression of interest' to the study coordinator to be screened for eligibility. To be included, participants will be required to have confirmed idiopathic PD, be able to participate in an outpatient exercise program including strength training and be willing to complete a falls calendar for 12 months after therapy. Participants will be excluded if they score less than 24 on the Mini-Mental State Examination (MMSE) [29], or if they have a rating greater than 4 on the modified Hoehn and Yahr scale [30] or if they are on major tranquilizers.

### Sample size

The sample size for the study was determined based on the falls rates and effect sizes obtained from previous trials on people with PD and the elderly [6,31,32]. For each sample size calculation, power was set at 80%, alpha set at 5%, and the drop-out rate was set at 15%. For falls, a sample size of 110 was thought to be required to detect a 20% difference between the groups, assuming a falling rate of 60% in the control group. For walking speed, a sample size of 74 was required to detect a 10% difference between the groups, assuming a mean (± SD) walking speed in the control group of 58.4 (12.0) m/min. For the "timed up and go" test, a sample size of 83 was thought to be required to detect a 10% difference between the groups, assuming a mean (± SD) timed up and go in the control group of 13.78 (2.99) sec. Thus, to ensure adequate statistical power for each of these outcome measures, the sample size for the study would optimally be 110 per group (total n = 330). Approximately 60 percent of control group participants are expected to fall between Test 1 (baseline) and Test 4 (12 months after therapy) with falls reduced to around 40% in the two active therapy groups.

### Interventions

#### Movement strategy training (MST)

All interventions will be delivered by therapists trained in the standard protocols. The MST group will attend two hours of physical therapy weekly in an outpatient setting for eight sequential weeks, in small classes of three or four participants. The MST group will follow a program based upon the principles of physical rehabilitation outlined in Morris et al., [13,33,34]. Table 1 summarises the content and dosage of the MST program with guidelines for therapists. Movement strategies emphasise task specific practice of everyday functional actions such as rolling over, standing up, walking, crossing obstacles and turning. These tasks are practised with strategies such as visual or auditory cues, mental rehearsal and movement planning, conscious attention during the task, and breaking the task into a sequence of smaller components. The program is tailored to movement impairments, activity limitations, and cognitive status and learning ability. An individualised home practice session of strategies to practise within the home or community will also be completed once a week. Structured falls risk education will also be provided each week, based upon the content of a booklet *Don't fall for it. Falls can be prevented! - A guide to preventing falls for older people *produced by the Australian Commonwealth Department of Health and Ageing [28]. This booklet contains information on topics such as medication, vision, footwear, regular activity and safe environments. A single home visit will be conducted by a trained therapist or nurse to check compliance with the therapy program.

**Table 1:** Movement strategy training protocol

**Principles of Movement Strategy Training **[13,33,34,44]

• Use visual, auditory or somatosensory cues to optimise timing and amplitude of actions, movements and their components
• Use attentional strategies such as focussing on key movement components, visualising correct amplitude or pattern, mental rehearsal, visualisation
• Break down complex movements into parts and focus on each segment in sequence
• Practise individual components of activities separately before incorporating into whole
• Incorporate cues and attentional strategies into functional tasks with reference to home environment.
• Individualise strategies, repetitions, and environmental context, with consideration to level of disability and functional difficulties
• For people with mild levels of impairment, consider practising dual tasking to promote motor learning. For those with advanced disease, avoid dual tasks

**Activities**
***Walking***
Walking with visual cues or attention strategies to correct step size to criterion length. Incorporate stopping and starting, with auditory cues if gait initiation difficulties.
***Turning***
Walking while turning, in either 'arc' or 'clock' (on the spot) patterns. Cues may include tape on floor, cue cards, photos, with attention to step size and placement.
Practise different turn magnitudes and turn activities relevant to home and community environment.
***Reaching in Standing***
Practice varied reaching activities in functional contexts, progressing to reach for objects of different weights and sizes at differing heights. Emphasise conscious attention to postural stability and position prior to reach.
***Sit to stand***
Modify chair height to assist or challenge patient, practise from different styles of chairs, chair height and compliances of chair and floor surface. Emphasise conscious attention to movement sequence in conjunction with amplitude, speed and flow of movement. Use visual cues such as photos or cue cards with key words.
***Transfer from chair to chair***
Practice transferring from chair to chair with the seats in different configurations, including at tables and in theatre rows. Encourage conscious attention to movement components in sequence and ensure safety. Visual and auditory cues may include cue cards with key words or pictures.
***Getting up from bed***
Emphasise normal timing and maintain momentum. Practise from same side of bed as person uses at home. Use assistive devices such as a bedpole where appropriate. Attentional strategies should focus on position in bed, sequence and speed and flow of movement. Cues could include cards with key words, photos or pictures.
***Protective stepping in standing***
Practise taking quick, large steps in different directions. Practise responding quickly to a verbal cue to step, or to a tug or push in a known or unknown direction. Encourage attentional strategies, such as focussing on visualising big and fast steps in the direction of loss of balance.
***Complex walking tasks***
Practice dual or multi-tasks, and obstacle course negotiation. Encourage mental preparation or movement visualisation, with pre-planning of the obstacle course and recognition of potentially difficult areas.

#### Progressive resistance strength training

The PST intervention group will attend a physical therapy program of equivalent length to the MST group; two hours weekly for eight weeks in an outpatient setting, in small classes of three or four participants. The PST program will include strengthening exercises for quadriceps, hip and trunk extensor muscles, hip abductors, calf, and ankle dorsiflexors, tailored to the individual's strength and functional ability. Table 2 summarises the content and dosage of PST with guidelines for therapists. Where possible, training is performed in functional tasks such as standing up from a chair, stepping up onto a step etc, using body weight, weighted vests and Thera-band^® ^to progress the resistance. Structured falls risk education will also be provided each week, in a manner identical to the MST group, using the same *Don't fall for it. Falls can be prevented! - A guide to preventing falls for older people *resource booklet [28]. An individualised home practice session of strengthening exercises will also be completed once per week. A single home visit will be conducted by a trained therapist or nurse to check compliance with the therapy program.

**Table 2:** Progressive Resistance strength training protocol

**2. Principles of Progressive Resistance Strength Training **[17,18,20,21,45]

• Safety is paramount. Ensure
○ correct execution of the movement
○ the required number of repetitions and sets is achieved
○ the required level of supervision or support is provided
○ participants advise therapists of any concerns or adverse symptoms
• All exercises will be steadily progressed, either by
○ increasing the number of repetitions (aim for 8-15)
○ increasing the weight or resistance (2% bodyweight increments)
○ altering starting position
○ increasing number of sets (to maximum of 3)
• Progression is guided by
○ the participant's ability to correctly perform the movement
○ the Modified Perceived Exertion scale (mRPE) [45] - when the exercise level is ≤ 5 on this scale
○ completion of 8-15 repetitions for 1-2 sets
○ clinical judgement of the therapist

**Exercises**
***Sit-to-stand***
Sit to stand from chair. Progress with variation of use of arms, vary height of chair and use of weighted vest.
***Trunk extension and rotation***
Seated, Thera-band^® ^loop under one foot, use both hands, (i) flex elbows until hands touch opposite shoulder, then (ii) rotate and extend trunk.
***Lateral pelvic hold/hitch***
Subject single leg stance on small step and hand support. Shorten or hitch so pelvis is horizontal and hold for count of 5 - 30 secs. Progression includes use of weighted vest.
***Step-ups***
Subjects stand in front of step with hand support nearby. Step up then return, first with right leading, then with left leading. Progression includes use of weighted vest.
***Heel raises***
Participants stand facing wall, toes approximately 40 mm from wall. Lean forearms on the wall, then push up onto the balls of the feet. Hold for count of 10. Progression includes use of weighted vest.
***Standing toe raises keeping balance***
Standing upright, with back close to wall for safety, raise one forefoot, hold, then down, followed by the other.
Progress to both feet together, then standing toes down on a wedge.
***Abdominals***
Subject sitting on edge of a chair with a back, with hands on opposite shoulders. Keeping back straight, and abdominals braced, lean backwards a short distance. Hold for count of 5 - 10.

#### Life-skills control group

The control intervention will be of equivalent duration to the MST and PST groups, delivered in small groups in outpatient settings over 8 weekly two hour sessions. Each session will be led by trained occupational therapists, physical therapists, speech pathologists or social workers, and include content such as relaxation, games, or communication activities. Guided discussion will also include topics such as the impact of PD on the individual and family, support and resources available, and fatigue management. None of the content will relate to walking, balance or falls risk education. A home session of reflection activities and relaxation practice will also be completed once per week.

### Outcome measures

**Figure 1:**
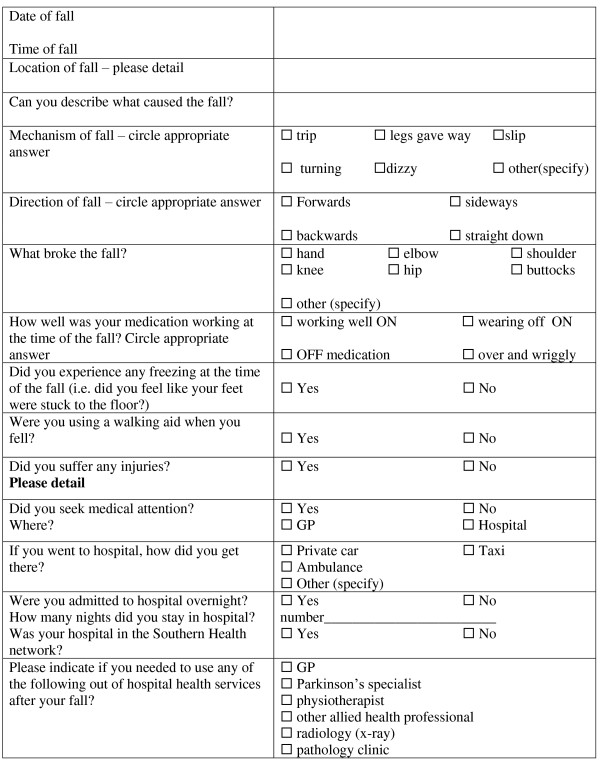
**Falls interview questions**.

All participants will be tested by trained blinded assessors before and after the 8 week interventions, and at 3 months and 12 months after completion of the intervention phase. Participants will keep a falls calendar for 12 months after therapy. Consistent with the recommendations of the Prevention of Falls Network Europe (PrOFaNE) group, a fall will be defined as "an unexpected event in which the participant comes to rest on the ground, floor, or lower level" [35]. Each time a participant falls, they will tick the date on the falls calendar and then telephone a falls hotline where they will be interviewed about the nature of the fall, circumstances, injuries sustained, healthcare required and other factors. The falls interview questions are given in Figure 1, and an example of a calendar page is provided as an additional file (see Additional File 1).

The three falls outcomes that are the primary outcomes are (i) the number of fallers in each group (ii) the number of multiple fallers in each group and (iii) the falls rate over 12 months in each group. Secondary outcome measures will include the number of injurious falls, defined as a fall requiring a visit to a health service provider. Additional secondary outcome measures include mobility, measured by quantifying walking speed using the 6m walking test and the Timed Up and Go Test [36]. Activity limitations will be quantified using the United Parkinson's Disease Rating Scale sections II and III [37]. Quality of life will be measured using the Parkinson's Disease Questionnaire-39 (PDQ39) [38] and EuroQol-5D [39]. Changes in health-related QoL will be measured using both a PD-specific measure (PDQ-39) and a generic utility instrument (Euroqol-5D) administered at baseline, immediately post-intervention, 3 months, and 12 months. Safety during the intervention phase will be assessed by time to first fall, and monitoring of adverse events related to therapy.

### Statistical analysis

Statistical analysis will be undertaken using IBM SPSS version 19.0 (SPSS Corp, Chicago, Ill, USA) and STATA 8 (Stata Corp, College Station, Tex., USA) statistical software. All analyses will be conducted on an intention-to-treat principle using all randomised participants. Demographic characteristics and baseline data will be summarised by descriptive statistics. For the primary outcome measures, the number of fallers and multiple fallers in each group will be compared by calculating relative risks. The number of falls and falls rate per person per year in the three groups will then be compared using negative binomial regression models. This approach takes into account all falls and adjusts for varying duration of follow-up [40,41]. As a safety measure, time to first fall during the intervention period will be analysed using Kaplan-Meier survival analysis, and comparisons between groups will be made using the Mantel-Cox log rank test. The continuously-scored secondary outcome measures at baseline and the follow-up appointments will be compared using analysis of covariance with baseline scores and intervention group entered as independent variables [42,43]. For categorical secondary outcome measures, relative risks will be used to analyse differences between the groups.

## Discussion

This RCT will be one of the first investigations of the effects of physical therapy interventions on falls in people with idiopathic PD. It will also be one of the first PD studies to collect detailed data on falls over a 12 month period, using a falls calendar coupled with telephone interviews. This will provide an accurate record of falls rates in a large cohort from a major metropolitan city. It will also provide data on the contributing factors to falls in people with PD and the injuries sustained and healthcare required. Such data are not currently available and will be of benefit to policy makers, healthcare professionals and people living with PD. Data are also being collected on impairments, disability and quality of life, allowing examination of the associations between these variables and falls frequency and severity in PD.

There are a number of limitations of the current trial. Participants will all be rated 0-IV on the modified Hoehn and Yahr Scale [30] and therefore the results cannot be generalised to those rated V, who are severely disabled. All measurements will be taken during the day at peak dose of the levodopa medication cycle and therefore the effects of medication cannot be separated from the effects of physical therapy and falls education. The response to strength training, strategy training or life-skills social interventions in the "off' phase of the levodopa cycle cannot be ascertained. Moreover the results can only be generalised to outpatient therapy services and may not be directly applicable to home or hospital care.

## Conclusions

This RCT will show the extent to which outpatient physical therapy programs combined with falls prevention education are effective for reducing falls and improving mobility and life quality in people with PD who live in the community. Moreover it will show whether strategy training or strength training is more effective than a control intervention for people with mild-moderately severe PD, when combined with education on falls prevention.

## List of abbreviations

PD: Parkinson's Disease; MST: Movement strategy training; PST: Progressive resistance strength training; RCT: Randomised controlled trial; PDQ39: Parkinson's Disease Questionnaire-39.

## Competing interests

The authors declare that they have no competing interests.

## Authors' contributions

MM, JM, FH, RI, HM, JW and AM conceived the idea for the study and participated in the design of the study as well as project management, data analysis, sample size calculations and data interpretation. AM and MD contributed to the design and assisted with subject recruitment and project management. MM, JM, HB, DK, MD and SS drafted the manuscript for submission and will be involved in compiling patient information and analyzing results. All the authors have read and approved the manuscript.

## Pre-publication history

The pre-publication history for this paper can be accessed here:

http://www.biomedcentral.com/1471-2377/11/93/prepub

## Supplementary Material

Additional file 1 **Falls Calendar**. An example of a page from the Falls Calendar. Click here for file
