# Supplementary material for: Falls and mobility in Parkinson's disease: protocol for a randomised controlled clinical trial
Source: BMC Neurol. 2011 Jul 31;11:93. doi: 10.1186/1471-2377-11-93 (PMC3160881; doi:10.1186/1471-2377-11-93)
Supplement: Additional file 1 — Falls Calendar. An example of a page from the Falls Calendar. [file 1471-2377-11-93-S1.DOCX]

**Supplementary File 1: An example page from the Falls Calendar**
